# Microalgal Phycoremediation: A Glimpse into a Sustainable Environment

**DOI:** 10.3390/toxics10090525

**Published:** 2022-09-06

**Authors:** Biswajita Pradhan, Prajna Paramita Bhuyan, Rabindra Nayak, Srimanta Patra, Chhandashree Behera, Jang-Seu Ki, Andrea Ragusa, Alexander S. Lukatkin, Mrutyunjay Jena

**Affiliations:** 1 Algal Biotechnology and Molecular Systematic Laboratory, Post Graduate Department of Botany, Berhampur University, Bhanja Bihar, Berhampur 760007, Odisha, India; 2 Department of Biotechnology, Sangmyung University, Seoul 03016, Korea; 3 Department of Botany, Maharaja Sriram Chandra Bhanja Deo University, Baripada 757003, Odisha, India; 4 Cancer and Cell Death Laboratory, Department of Life Science, National Institute of Technology Rourkela, Rourkela 769001, Odisha, India; 5 CNR-Nanotec, Institute of Nanotechnology, Via Monteroni, 73100 Lecce, Italy; 6 Department of Biological and Environmental Sciences and Technologies, Campus Ecotekne, University of Salento, Via Monteroni, 73100 Lecce, Italy; 7 Department of General Biology and Ecology, N.P. Ogarev Mordovia State University, Bolshevistskaja Str., 430005 Saransk, Russia

**Keywords:** microalgae, bioabsorption, heavy metals, pollution, phycoremediation

## Abstract

Microalgae are continually exposed to heavy metals and metalloids (HMMs), which stifles their development and reproduction due to the resulting physiological and metabolic abnormalities, leading to lower crop productivity. They must thus change their way of adapting to survive in such a hostile environment without sacrificing their healthy growth, development, reproductive capacity, or survival. The mode of adaptation involves a complex relationship of signalling cascades that govern gene expression at the transcriptional and post-transcriptional levels, which consequently produces altered but adapted biochemical and physiochemical parameters. Algae have been reported to have altered their physicochemical and molecular perspectives as a result of exposure to a variety of HMMs. Hence, in this review, we focused on how microalgae alter their physicochemical and molecular characteristics as a tolerance mechanism in response to HMM-induced stress. Furthermore, physiological and biotechnological methods can be used to enhance extracellular absorption and clean up. The introduction of foreign DNA into microalgae cells and the genetic alteration of genes can boost the bio-accumulation and remediation capabilities of microalgae. In this regard, microalgae represent an excellent model organism and could be used for HMM removal in the near future.

## 1. Introduction

Heavy metals and metalloids (HMMs) play a key role in regulating the growth of organisms, as they influence their stress-response mechanisms. They are responsible for triggering many stress responses, including several biochemical responses in terrestrial plants as well as algal biosystems [1]. However, HMM excesses are among the most significant contaminants exerting a negative impact on crop growth and yield. In recent years, HMMs have become major problems, with a huge impact on nutritional, environmental, and ecological conditions [2]. As a result of various natural processes, such as weathering and erosion, and human actions, such as mining and industrial activities, wastewater, agrochemicals, and automobile exhaust gases, the accumulation of HMMs, such as lead, mercury, arsenic, and cadmium, in water bodies has been increasing day by day [3]. HMMs are responsible for water pollution and hamper the ecosystem. HMMs are present in two forms: immobilized and soluble. Compared to the soluble form, immobilized HMMs are more hazardous to plants and microalgae [4]. 

Metals with density values greater than 5 g/cm^3^ are classified as HMMs [5]. HMMs actually make up 53 of the 90 naturally occurring elements [6]. Water-soluble HMMs include manganese, iron, cadmium, arsenic, lead, mercury, etc., of which cadmium, lead, copper, molybdenum, nickel, and zinc are the ones that cause the most damage to crops [7]. Although some HMMs act as micronutrients at low doses, at higher concentrations they lead to growth inhibition and metabolic disorders [8]. Among the 17 HMMs important for living organisms, molybdenum, iron, and manganese are essential micronutrients, while cobalt, zinc, copper, nickel, vanadium, tungsten, and chromium are the trace metals with varying degrees of relevance [9]. On the other hand, mercury, silver, cadmium, antimony, uranium, and lead are not nutrients, and are deemed to be more or less harmful [10]. Excessive concentrations of various metals in the soil, such as nickel, cadmium, chromium, zinc, and copper, can harm natural and terrestrial ecosystems [11].

HMMs cause an oxidative imbalance in organisms by generating numerous free radical species that exert a negative effect, leading to altered physico-biochemical responses with consequent induction of cell death [12]. Free radicals interact with biomolecules and replace their essential metal ions, thus causing impairment in many physiological processes, such as photosynthesis and cell growth [13]. Tolerance and removal of potentially toxic elements depend on the binding mechanism. Using algae to absorb HMMs in contaminated waters is a low-cost and environmentally friendly approach that has piqued the scientific community’s curiosity [14,15,16]. Many algae have a high capacity to absorb metals, and there is a lot of promise in employing them to clean up wastewater. Metal absorption entails binding to internal ligands as well as to the cell surface. The amount of metal adsorbed is many times greater than the intracellular concentration of the metal. The carboxyl group is crucial for binding the metal. Metal absorption is affected by the concentrations of metal and biomass in the solution, anions, cations, temperature, pH, and the metabolic stage of the organism. Based on these premises, in this review, we focused our attention on the sources of HMMs, their interaction with the algal biosystem, the physico-biochemical responses against these elements of stress, and the mode of detoxification.

## 2. Sources of Heavy Metal and Metalloids

**Figure 1:**
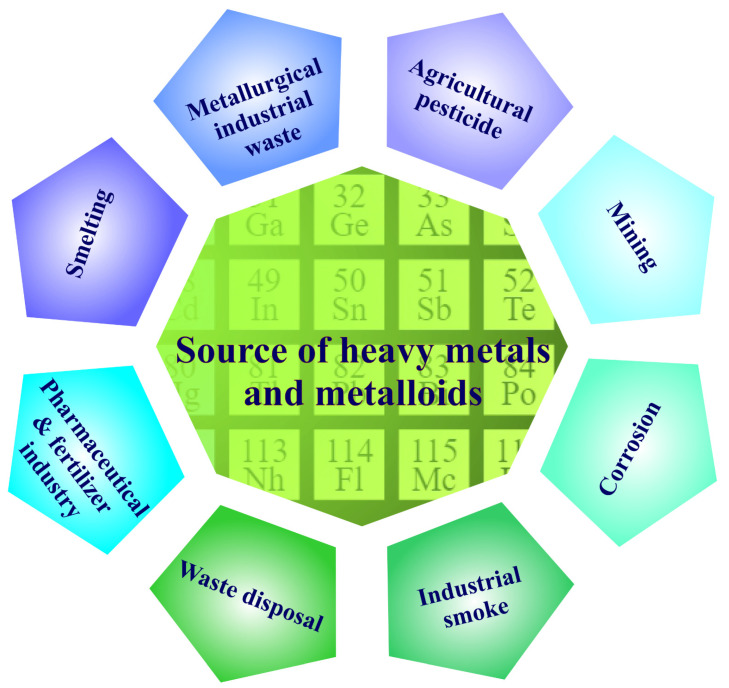
Main sources of heavy-metal and metalloid contamination.

HMM concentrations in the soil are linked to geochemical and biological cycles, as well as anthropogenic factors, such as industrial, agricultural, and wastewater treatment activities [17,18]. Heavy metal contamination is generated by a variety of natural and man-made processes, and it represents a serious environmental threat because of the toxicity, environmental persistence, and bio-acquisitive nature of HMMs. Geogenic, industrial, agricultural, pharmaceutical, domestic, and atmospheric sources have all been identified as sources of HMMs in the environment. Foundries, mines, and smelters, for example, are particularly significant sources of pollution [19]. The main sources that can lead to HMM contamination are shown in Figure 1.

### 2.1. Water

Aquatic system pollution caused by HMMs is a big global concern [20]. Heavy metal poisoning of water is basically unavoidable in some regions due to natural processes (e.g., rock erosion) and anthropogenic activity (industrial, agricultural, and domestic effluents) [21]. For instance, vegetables cultivated along the banks of rivers that flow through cities are irrigated by them. HMMs have been found in these rivers’ waters, as well as industrial effluents, sewage, and sludge, and automobile emissions have poisoned the majority of these areas. As a result, HMMs are likely to infect crops grown on such soils, rendering them unfit for human consumption [22]. HMMs such as cadmium, lead, and copper, are commonly found in high amounts in mining, electroplating, paint, and chemical wastes. These elements, at concentrations higher than those physiologically necessary for plants, can induce toxicity in them, as well as in food chains, thus also representing a problem for human health. Microalgae serve an important role in both marine and freshwater ecosystems by giving nutrients and energy to all living organisms in water bodies, as well as lowering HMMs concentrations [23].

### 2.2. Air

Significant efforts have been made in recent decades to reduce air pollution. Due to their effects on human health, plants, aquatic life, and materials, particulate matter (PM) from aerosols has attracted a lot of attention. Metals can be absorbed by vegetables from the soil, as well as deposited on parts of crops exposed to polluted air [24]. HMMs generated by industry and automobiles can settle on the surface of vegetables during their production, transport, and marketing. 

### 2.3. Soil

The breakdown of polluted plant components in the soil, combined with reduced bioabsorption and bioaccumulation, causes the redistribution of HMMs in the soil [25]. The presence of HMMs in the soil depends on their chemical structure, which determines their solubility and therefore their mobility and biological accessibility. HMM pollution in the soil is one of the most serious dangers to water and soil resources, and therefore to human health. Concentrations as high as 1000 ppm (0.1%) are toxic to plants and microalgae [26]. Water-soluble HMMs can be found in significant quantities in plants and microalgae due to their ability to be absorbed [27]. As expected, plants growing on farmland near dumpsites bioaccumulate more metals than those grown on distant farmland [28]. The selection of plant and microalgal species is fundamental in the development of remediation approaches (e.g., decontamination and stabilisation), especially in low- and medium-pollution areas [26,29].

## 3. Sources of Toxic Heavy Metal and Metalloid Pollution

HMMs, which are discharged in large quantities from a variety of chemical processes, can be found in high concentrations as effluents in areas adjacent to industrial sites. Industrial waste released into the environment without proper treatment is the main cause of HMM contamination and related toxicity [30,31]. Processes such as electroplating, conversion-coating, milling, and anodizing produce large quantities of wastewater containing HMMs such as nickel, lead, chromium, cadmium, copper, platinum, silver, zinc, vanadium, and titanium [32]. Furthermore, leftovers from printed circuit board production continue to be a source of industrial effluents containing HMMs with significant amounts of copper, tin, nickel, and lead. Inorganic paint manufacturing industries also generate effluents containing chromium and cadmium derivatives. In addition to these, frequent industrial operations, such as aquaculture, intensive livestock production, and energy production, are also responsible for the release of wastewater containing high concentrations of HMMs; for example, cadmium, copper, nickel, lead, chromium, and zinc. With ever-increasing HMM contaminations and related toxicity, detoxification procedures must be adopted to prevent this type of pollution and sustainable environmental development. In this context, the exploitation of a biosorbent, such as algal biomass, would be ideal and could be employed for effective elimination of HMMs [33].

## 4. Mechanism of Heavy Metal and Metalloid Removal Using Microalgae

**Figure 2:**
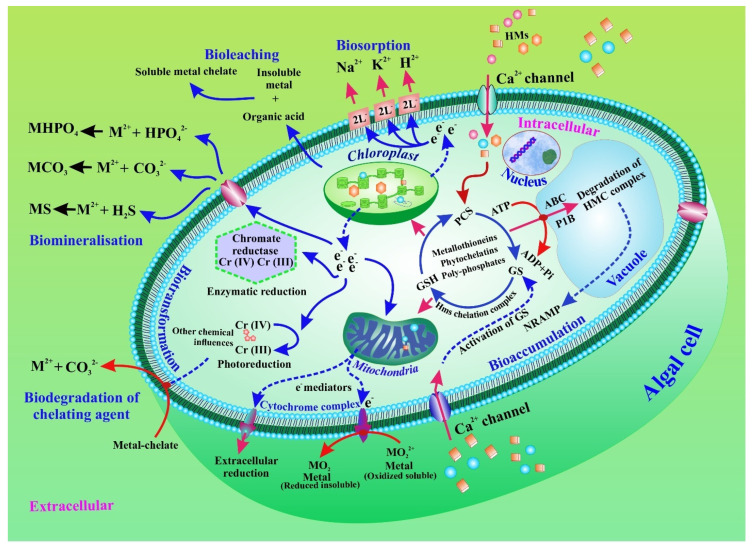
Bioremediation of heavy metals and metalloids in microalgae through accumulation and intra- and extracellular biotransformation. Biotransformation, bioleaching, and biomineralization are also displayed.

Microalgae are present in both marine and fresh water, as well as in polluted areas [34,35,36,37,38,39]. For the elimination of HMMs, both extracellular and intracellular phycoremediation techniques could be used. The main pathways involved in the elimination of HMMs are illustrated in Figure 2. The figure also shows that the interactions between metals and algae have a consequence for bioremediation. HMMs are metabolized intracellularly in microalgae by the accumulation and bio-transformation of heavy metals. The algal cell can remediate heavy metals extracellularly. The heavy metal is facilitated by metal transporters in the microalgal cell membrane. Bio-sorption of various negatively charged functional groups is used in the extracellular mechanism. Moreover, biotransformation, bioleaching, and biomineralization also play a critical role in the bioremediation process. Furthermore, the linked metal chelate is involved in the biodegradation of the chelating agent.

### 4.1. HMM Intake and Interactions in Microalgae

HMMs are the main concern in the current scenario [40]. Metals such as zinc, iron, nickel, copper, cobalt, manganese, and molybdenum are crucial for cellular metabolism. These metals are also found in metalloproteins, which are involved in a variety of cellular processes, including electron transport and protection from reactive oxygen species [41,42]. Concentrations of HMMs in the cell range from nanomolar to femtomolar, and the metal stoichiometry also differs among species [43]. Metal adsorption and transportation across the cell membrane normally occur in two stages of the uptake process, and the rate of transport is thought to be limited [44]. In a metabolism-independent process, metal ions are adsorbed to the cell wall by contact with the functional groups of important structural molecules, such as polysaccharides and proteins [45]. Subsequently, the metal ions can enter the cell membrane via active transport by binding to ion carriers or low molecular weight thiols, such as cysteine [46].

### 4.2. Bio-Removal of Extracellular HMMs by Microalgae

Biosorption of HMMs is a physicochemical characteristic of the microalgae cell surface that is able to bind to heavy metal ions in solution, without relying on cellular metabolism. The uptake of HMMs in living microalgae occurs by biosorption in the cell wall or through the extracellular polymeric substances (EPS) produced by the microalgae in response to stress. The biosorption of HMMs into the EPS is a metabolism-dependent process. Microalgae can regulate EPS synthesis in response to metallic stress, and they can also tune the characteristics of these biopolymers as needed [47,48].

#### 4.2.1. Microalgae Cell Wall Structure and Composition Play a Critical Role in HMM Biosorption

The cell wall acts as a barricade between the intracellular compartment and the outer environment [49]. The cell wall is made up of multifunctional macromolecules, such as carbohydrates, lipids, and proteins, which have several negatively charged functional groups on their surfaces, such as carboxyl, amino, hydroxyl, sulphate, phenol, sulfhydryl, phosphate, and so on [50]. The outer layer of the cell wall is the first participant in the elimination of HMMs because these negatively charged groups allow ions in the surrounding environment to bind [51]. When examining mechanisms of biosorption, it is crucial to comprehend the characteristics, structure, and composition of the cell wall [52]. Furthermore, other physicochemical factors, such as temperature, pH, other ions, and adsorbent ratio, also regulate the modality and efficacy of HMM removal [53].

In recent years, the most commonly employed microalgae strains in phycoremediation come from the phylum Chlorophyta; in particular, from the genera *Chlorella* and *Scenedesmus* [54]. The sensitivity and effectiveness of microalgae biosorption vary by genus and species, even under identical operating conditions [23]. For example, *C. sorokiniana* and *S. obliquus* grew differently in media polluted by copper(II), cadmium(II), lead(II), and chromium(VI) because of the different compositions and structures of their cell walls [55]. Within a phylum, the cell membrane can vary in complexity, from a simple lipid bilayer with peripheral and integrated proteins to a cap of glycolipids and glycoproteins enveloping the outer cell surface, as in *Dunaliella* and *Isochrysis* species. Complicated multilayer structures with additional intracellular material in vesicles can be found in dinoflagellates, cryptophytes, and euglenophytes species. They have both extracellular and intracellular material coupled with the cell membrane. Differences in the composition of the cell wall are also possible among species of the same genus; for example, *C. vulgaris* has an innermost layer [56], while *C. zofingiensis* and *C. homosphaera* have both an internal and an external layer, as well as a trilaminar form of the outer layer [57]. However, in *C. trilaminar*, sporopollenin forms the outermost layer, the middle layer consists of chitin and mannose-like polysaccharides, and the inner layer is composed of a phospholipid bilayer [58].

#### 4.2.2. Physicochemical HMM Interactions and Role of Microalgal Cell Surface

Understanding how heavy metal ions interact with the cell surfaces of microalgae is difficult due to the complexity of the cell surface. A number of chemical and physical interactions have been documented. Chelation and complexation of HMMs with active groups in the cell wall are the main mechanisms involved. Ions, such as calcium, sodium, magnesium, and potassium, can be reversibly replaced in solution by other harmful HMMs via an ion-exchange mechanism on the surface of microalgae cells [59]. Physical factors, such as Van der Waals and electrostatic interactions, can influence the physical adsorption mechanism of the metal-binding onto the cell surface. In addition, microprecipitation is a process related to both active and passive metal absorption pathways [60].

#### 4.2.3. HMM Interactions with Extracellular Polymeric Compounds

EPS are high-molecular-weight extracellular biopolymers produced by a variety of microorganisms, including microalgae. Proteins, lipids, nucleic acids, sugar, humic compounds, and other inorganic extracellular compounds that bind to carbohydrates are classified as EPS [61]. EPS soluble in media (SL-EPS), EPS connected to the cell wall or loosely bound EPS (LB-EPS), and tightly bound EPS (TB-EPS) are the three basic types of microalgal EPS [47]. The presence of harmful pollutants, such as toxic metals, is usually a threat to microalgae in aquatic habitats. EPS production is an adaptive process utilized as a self-defence strategy [62].

In general, when a metal is intercepted, EPS production increases. For example, Yu and colleagues found that, following cadmium exposure, production of LB-EPS by *C. reinhardtii* increased considerably [63]. Similarly, Li et al. recently showed that under Cd(II) and lead(II) stressors, *C. reinhardtii* produced more EPS [64]. In addition, an increase in EPS yields in copper enriched *Chlorella* sp. cultures indicated that EPS, rather than intracellular chelation, is responsible for copper absorption [65]. However, by comparing EPS-free and EPS-coated *C. pyrenoidosa* cells, EPS has been shown to improve adsorption capacity, minimize intracellular accumulation, and increase As ion tolerance [66]. EPS appears to have the ability to build an extracellular defensive barrier on the cell wall surface, consequently preventing HMMs from causing harm to the intracellular environment. [47]. Furthermore, EPS has a large number of charged hydrophobic groups that are responsible for dynamically binding to HMMs [66]. Hence, metal biosorption into the EPS can be related to the characteristics of the cell surface and of the functional groups [67]. The interactions that can control the biosorption of HMMs into microalgal cells are illustrated in Figure 2.

### 4.3. Bioaccumulation Mechanisms of HMMs in Microalgae

Bioaccumulation, unlike biosorption, is a metabolic mechanism in which intracellular accumulation of HMMs occurs through passive and/or active transport channels across the cell membrane [68]. It consists of two stages: first of all, there is a rapid, passive, and non-specific absorption of metal ions on the cell wall; after bioabsorption, passive and/or active transport to the cytoplasm occurs across the plasma membrane and cell wall [23]. For example, the absorption of cadmium(II) by the green algae *Tetraselmis suecica* is a biphasic process aided in the initial phase by the adsorption of proteins or polysaccharides, followed in the second phase by an energy-dependent accumulation of the HMMs in the cytosol [69]. Furthermore, when the extracellular concentration of the metal is significantly higher than the intracellular concentration, cations can be transported into the intracellular compartment by negatively charged groups on the cell surface via active transport across the plasma membrane after binding to thiol molecules, mainly cysteine [5]. Histidine, glutamate, and proline are some of the additional amino acids that help with the metal chelation and detoxification [23]. Since most HMMs are hydrophilic, they are transported across the plasma membrane by particular metal transporters [5].

#### 4.3.1. Metal Transporters in Microalgae Cell Membrane

Metal transporters play a crucial role as they represent the first line of defence in terms of regulating osmotic balance. In addition, they regulate the intracellular absorption of ions critical for micronutrient homeostasis and mitigate the subsequent negative effects of non-essential HMMs [70]. The participation of several membrane transporters has been reported in a variety of microalgae species [71]. For example, entrance and outflow of metal ions in *C. reinhardtii* can be mediated by natural resistance-associated microphage proteins (NRAMP), the Fe-transporter (FTR), Zrt-Irt-like proteins (ZIP), and the copper transporter (CTR), ensuring the movement of HMMs from the extracellular surface to the cytosol [72]. These transporters have also been discovered in the vacuole membrane, and they serve the same purpose as the assimilative transporters. Through the efflux of active metal ions into the extracellular environment, members of the cation diffusion facilitator (CDF), FerroPortiN (FPN), P1B-type ATPases, and the calcium (II)-sensitive cross-complementer 1/Vacuolar iron transporter 1 (Ccc1/VIT1) lower the metal content in the cytoplasm (Figure 2). Once the metal concentration exceeds the cellular requirement, or when the metal peptide complex begins to interfere with cell metabolism, the metal transporters regulate the metal concentration in the cell [73]. With limited reports available about metal transporters in algae, further studies are needed to understand the exact mechanisms behind this process.

#### 4.3.2. Pathways of Intracellular HMM Detoxification in Microalgae

Microalgae employ various processes to maintain intracellular ion concentrations and shield the cell from non-essential metals [74]. These include modification of plasma membrane permeability and cell wall function, stimulation of phytochelatin synthase, creation of HM-metallothionein and HM-polyphosphate complexes, compartmentalization into organelles, and activation of metal efflux mechanisms, allowing the maintenance of intracellular ionic homeostasis [75].

##### Chelation by Metallothioneins and Phytochelatins

Metallothioneins and phytochelatins are metal-binding proteins mainly responsible for maintaining a stable intracellular metal concentration [76]. Phytochelatins are small peptides that can be categorized into two classes: gene-encoded proteins, such as class I and II metallothioneins, and enzymatically produced polypeptides, such as class III metallothioneins [77]. Class II metallothioneins are a cysteine-rich superfamily of proteins found in the cytosol and have a low molecular weight of 6–7 kDa. *Aureococcus*, *Chlorella*, *Nannochloropsis*, *Ostreococcus*, *Symbiodinium*, and *Thalassiosira* are microalgae with the most known metallothioneins so far [78]. Microalgae have the potential to produce novel types of metallothioneins, as they can survive in heavy metal-contaminated environments.

Phytochelations (PCs) can be produced enzymatically, rather than genetically by microalgae. PCs are thiol-containing peptides consisting of three amino acids: cysteine (Cys), glycine (Gly), and glutamate (Glu), and typically have a (γGlu-Cys)*_n_*-Gly structure, with 2 < n < 10. Metal binding is accomplished by the sulfhydryl group of the cysteine molecule. Production of -Glu-Cys by glutamylcysteine synthetase (GCS) is the first biosynthetic step. Subsequently, glutathione synthetase (GS) catalyses the synthesis of glutathione (GSH). Finally, another GSH molecule transfers -Glu-Cys to obtain (-Glu-Cys)_2_-Gly [79]. When intracellular metal concentrations are low, GSH is the main ligand; however, when high amounts of metals are absorbed, the PCs are responsible for their elimination [76]. Several studies have documented that the formation of class III MTs is responsible for detoxification in microalgal strains. In *Chlorella fusca*, MTs are formed after exposure to cadmium(II) ions [80]. Other studies aimed to clarify the biosynthesis of PCs when microalgae are exposed to HMMs. For instance, Gomez-Jacinto et al. discovered the formation of mercury–PC complexes in *C. sorokiniana* exposed to mercury [81], in copper(II)-treated *Stichococcus bijugatus*, and in lead(II)-treated *Stichococcus bacillaris* [82]. In addition, cadmium(II) was found to be the most powerful stimulator of PC synthase in *Chlamydomonas* species [64]. On the other hand, zinc was found to be the most potent inducer of PC synthesis in *Dunaliella* species [83]. A recent study discovered that GSH is the most abundant non-protein sulfhydryl molecule in *D. salina* [84]. Exposure to arsenic(V) and arsenic(III) led to the synthesis of PCs, implying that they are involved in As detoxification.

##### Chelation by Polyphosphates

Orthophosphate polymers (polyP) are abundantly found in both prokaryotic and eukaryotic cells. Several studies in algae revealed that polyP bodies accumulate in acidocalcisomes, which are formed primarily in granules of particular vacuoles in the trans-Golgi [85,86]. PolyPs are also present in the cytoplasm, nucleus, endoplasmic reticulum, mitochondria, and cell wall [87]. In *C. reinhardtii,* the metabolism of polyP can be regulated by acidocalcisome membrane transporters through enzymatic exopolyphosphatase reactions [88]. PolyPs are involved in a variety of functions, including HMM sequestration and detoxification [87]. The production of polyP also helps in the collection and storage of HMMs [89]. Indeed, the critical role of acidocalcisomes and polyPs in supporting cellular homeostasis of essential ions can be further expanded in relation to the bioaccumulation of hazardous HMMs [75].

#### 4.3.3. Compartmentalization of HMMs in the Vacuole, Chloroplast, and Mitochondria

Sequestration of the MT-HMM complex in specific cell organelles, such as mitochondria, chloroplasts, and vacuoles, leads to the formation of metal bioaccumulation pathways and tolerance mechanisms. Transmission electron microscopy (TEM) with additional techniques and accessories, such as energy-dispersive X-ray spectroscopy (EDS), electron spectroscopic imaging (ESI), electron energy loss spectroscopy (EELS), and atomic force microscopy (AFM), can be used to study HMMs and their complexes (polyP-HMMs, MTs-HMMs, PCs-HMMs) [73]. Vacuolar assortment has been recognized as an essential component of HMM detoxification in several plant species [90]. On the other hand, metal sequestration has been discovered in a variety of cell organelles. A study discovered electron-dense black spherical entities in the vacuoles of *Pseudochlorococcum typicum* subjected to lead ions by using TEM examination [91]. Moreover, by using TEM, EELS, and ESI, the accumulation of chromium(IV) in a chromium–iron–oxygen complex and enhanced vacuolation inside *Micrasterias denticulata* cells were discovered [92]. In contrast, the chloroplast was the primary storage location for PC–cadmium(II) complexes in *C. reinhardtii* [70]. Similarly, a study revealed that the chloroplast of *Euglena gracilis* contains more than 60% of the accumulated cadmium(II) [93]. Another study discovered that the intracellular copper (II) in the thylakoids and pyrenoids of *O. nephrocytioides* also accumulates HMMs [94]. Furthermore, Mendoza-co et al. found that cadmium(II) and class III MTs-cadmium(II) complexes accumulated in the mitochondria and chloroplast of *E. gracilis* [93]. All the above studies suggest that microalgae may be the most important organisms for eliminating HMMs.

### 4.4. Biotransformation and Mitigation of HMMs by Microalgae

The mechanism by which endobiotic or xenobiotic compounds are converted into molecules that differ in activity, excretability, and toxicity is referred to as biotransformation (detoxification vs toxication) [95]. Although biotransformation may refer to a series of detoxification mechanisms, microalgae mainly use enzymatic and biochemical reactions to transform poisonous HMMs into harmless species.

#### Role of Enzymes in the Biochemical Transformation of HMMs

The enzymatic biotransformation of HMMs is described as the chemical transformation of a highly hazardous form into a less dangerous form through oxidation and reduction processes. HMMs cannot be destroyed, but they can be converted into an inorganic complex with minor harmful effects by changing their oxidation state. A few investigations focused on the involvement of oxidoreductase enzymes in HMM detoxification by microalgae. Arsenate reductase, mercuric reductase, and chromate reductases are the most common redox enzymes found in microalgae [96]. *C. vulgaris* has the ability to convert chromium(VI) to chromium(III) through an series of enzymatic reactions catalyzed by chromate reductase [97,98]. In addition, *Selenastrum minutum*, *Galdiera sulphuraria*, and *C. fusca* can catalyze the bio-transformation of Hg^2+^ into elemental mercury and metacinnabar (Mercury(II) sulfide) via the mercuric reductase enzyme [99]. *C. reinhardtii* also has arsenate reductase to detoxify arsenic [100].

Microalgae use biochemical mechanisms to mitigate HMMs during the phytoremediation process. The reduction of chromium from the hexavalent oxidation state to the trivalent form is catalysed by the transfer of electrons from the reduced form of GSH [98]. In addition, various detoxifying pathways reduce the toxicity of inorganic arsenic [101]. Some microalgae species appear to be capable of converting arsenic(V) to arsenic (III). A study revealed that after 72 h of exposure to arsenic(V), 32% of the total intracellular arsenic(V) concentration was transformed into arsenic (III) [102]. *C. aciculare* also converted some arsenic(V) in the cell medium to arsenic (III) [103]. With the use of oxidase and *S*-adenosylmethionine, the arsenic(V) was reduced to arsenic (III) and then methylated to monomethylarsonate (MMA(V)). The MMA was transformed to dimethylarsinate (DMA(V)), with subsequent reduction to DMA(III). Arsenic can also be reduced to arsenolipids, arsenosugars, arsenobetaine, and arsenoribosides [104].

## 5. Perspectives for Improving the Phycoremediation Process: The Unsoiled Sustainable Future

To date, several researches have proved the technical and economic feasibility of employing microalgae in the remediation of HMMs. To improve extracellular uptake and selectivity for a target metal, physicochemical and biotechnological techniques can be applied. In addition, techniques can be employed for boosting intracellular bioaccumulation capacity, as well as biotransformation and mitigation capabilities.

### 5.1. Enhancing Biosorption Capability and Selectivity through Cell Manipulation: The Emerging Era of Phycoremediation

Biosorption of HMMs occurs mostly at the cell surface. Alteration of cell wall composition and physicochemical features can increase biosorption of HMMs through increased interaction with heavy metal ions.

#### 5.1.1. Physicochemical Approaches

##### Chemical Pre-Treatment and Cell Surface Functionalization

Many chemicals can be employed as pre-treatment reagents to alter the physicochemical properties of the cell wall, including the removal of surface contaminants and blocking ions, thus exposing the binding sites and enhancing the biosorption capacity [105]. Inorganic salts (sodium carbonate and sodium chloride), organic solvents (toluene, alcohol, and acetone,), alkalis and acids can be used as such agents [106].

The exposed functional groups have a significant impact on the cell surface attraction for HMM species. The biosorption capabilities of cadmium(II), lead(II), nickel(II), zink(II), and copper(II) in *Neochloris minuta* and *N. alveolaris* have been evaluated based on biomass, composition, and type of hard or soft metal acid [107]. Biosorption can be controlled by changing the type and quantity of functional groups on the cell surface. Chemical processes that add active functional groups (binding sites) or suppress functional groups that have a detrimental impact on biosorption can be used to functionalize the cell surface [108].

#### 5.1.2. Bioengineering of Cell Surface Approaches: The Effective Technology of the Future

**Figure 3:**
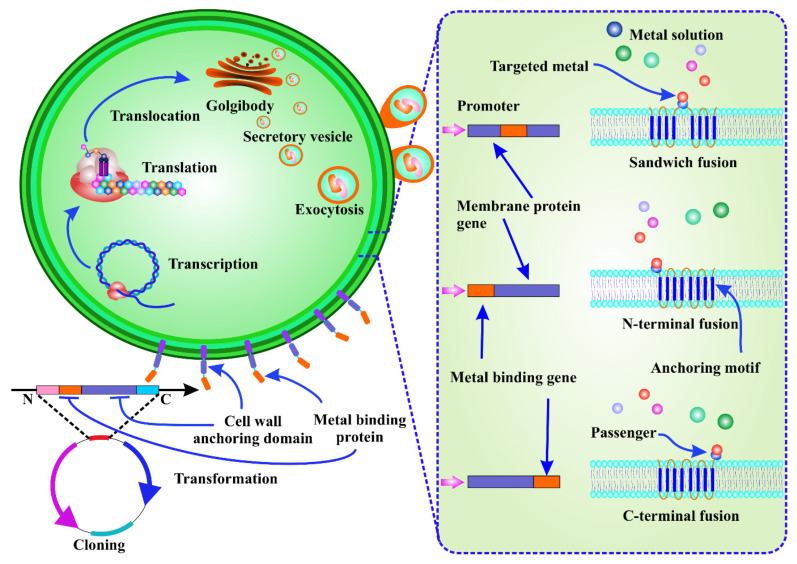
Cell surface engineering for the biosorption of a target metal. It involves the expression of a passenger protein on the cell surface by translational fusion with a carrier protein, which allows the passenger protein to be exported to the cell membrane and anchored to the cell surface. After transcription, translation, and translocation, the metal-binding peptide can be seen as a fusion of an anchor protein.

Although adsorption-based techniques have significant potential for HMM absorption, the lack of selectivity for targeted metal absorption from heterogeneous metal ion solutions is the main drawback associated with this process [109]. By changing the metallosorption characteristics of cell surfaces to improve adsorption selectivity for target metal species, the bioengineering of living microorganisms has enabled the development of novel biosorbents [110]. Cell surface engineering, also known as cell surface display, is a molecular approach that involves the expression of functional proteins of interest (passengers) on the cell surface by translational fusion to an anchor protein. As a result, the passenger protein is able to cross the cell membrane [108]. Artificial proteins with new functions, such as strong metal-binding affinity, can be employed to remove HMMs using genetic and protein engineering (Figure 3) [111].

Mechanistically, the target metal-binding protein or peptide-coding DNA extracted from genomic or plasmid DNA using full sequence synthesis or PCR amplification is induced in the host by protein fusion. After translocation, transcription, and translation, the metal-binding protein/peptide is seen to be integrated as a fusion of an anchor protein. Passenger and carrier proteins are enclosed in secretory vesicles that pass through the cell membrane and anchor the passenger proteins to the cell wall surface [110]. With limited reports available on bioengineered microalgal cells, He et al. demonstrated that transgenic *C. reinhardtii* (2AMT-2) expressing a membrane-anchored MT polymer had a high mercury (II) removal capacity when combined with sonication over a broad pH range of 2–9, which allowed them to solubilise mercury from solids and sediments [112]. Overexpression of metal-binding proteins on the microalgae cell surface can provide numerous benefits, such as reduced processing time and higher numbers of ligands on the cell surface. Instead of collecting the metals inside the cell, the metal adsorbed on the outer surface can simply be retrieved with a mild pickling reagent. In addition, the biosorbents can be recycled and are cost-effective. Finally, as surface adsorption is not affected by metabolism, dead biomass can be utilised.

### 5.2. Microalgae Engineering for Intracellular Recovery of HMMs

To improve the intracellular uptake of HMMs, the genetic modification of genes encoding metal membrane transporters, high-affinity HMM-binding proteins, such as genetically encoded chelators, enzymes that catalyse the reduction of toxic metals by redox transformations, and enzymes that scavenge reactive oxygen species, can all be used [12]. In fact, gene overexpression and the development of transgenic algae through the introduction of foreign DNA into microalgae cells can improve the bioaccumulation potential of microalgae [113].

#### Metal-Transporter Transition in Microalgae and Molecular Manipulation for AA and PC Biosynthesis

Phycoremediation requires the identification of microalgal genes that express metalloregulatory proteins. Metal transporters are important in the interactions of microalgae with the surrounding environment. They also serve as a second line of defence that controls metal homeostasis at the cellular and subcellular level [72]. HMM phycoremediation through genetic alteration of metal transporters can reduce HMM-related toxicities while also improving the removal of harmful metal ions from the cytosol to intracellular compartments. Overexpression of the metal tolerance protein (MTP) in *C. reinhardtii* increased tolerance to cadmium(II) toxicity as well as bioaccumulation efficiency through cadmium(II) transport and storage in vacuoles [114]. In addition, a cadmium and zinc transporter (AtHMA4) in *C. reinhardtii*, both as a full-length protein and as a *C*-terminal tail, was exploited to further promote zinc (II) and cadmium(II) ion bioaccumulation and internalization [115]. The arsenic bioremoval ability of an ACR3-modified *C. reinhardtii* strain was improved by transforming the wild-type strain with *Agrobacterium tumefaciens*, which used the pARR1 construct carrying a synthetic, optimised ACR3 gene from *Pteris vittata*, allowing for the removal of 1.5 to 3 times more arsenic than the wild-type strain [116].

The metal chelator is primarily responsible for the bioaccumulation of HMMs within the cell. The most successful strategy to enhance the bioaccumulation of HMMs in microalgal cultures appears to be the genetic alteration of the production and metabolism of specific AAs and PCs. Despite the great potential for AA and PC production to play a role in HMM tolerance and detoxification, only a few microalgae mutants are currently available for this purpose. Compared to the wild type, overexpression of the HISN3 gene (coding for phosphoribosylformimino-5-aminoimidazole carboxamide ribonucleotide isomerase) in *C. reinhardtii* results in high Ni tolerance and modest increase in histidine accumulation [117]. In addition, expression of the mothbean P5CS (Δ^1^-pyrroline-5-carboxylate synthase) gene in *C. reinhardtii* causes proline accumulation and increases resistance to cadmium(II) ions [12,118]. Furthermore, *C. reinhardtii* cells articulating foreign class II MTs showed greater efficacy of biosorption when exposed to cadmium(II) ions [119].

## 6. Conclusions and Future Perspectives

EPS-complexation, extracellular biosorption, intracellular bioaccumulation and compartmentalization, biomethylation, enzymatic reduction, and volatilization are all methods that green microalgae can adopt for eliminating harmful HMMs. Phycoremediation of HMMs is still a lab-scale process. Nonetheless, further research on underlying mechanisms can be considered to increase efficiency, selectivity, and reduce processing costs. Physicochemical and molecular changes in the cell surface of algae have recently been shown to be useful approaches for improving the effectiveness of bioremediation. Hence, bioengineering the cell surface with novel chemical agents to induce favourable cell surface characteristics could help in the process of biosorption. On the other hand, only a few cases of genetic manipulation of microalgae for the elimination of HMMs have been reported. More emphasis needs to be placed on the genetic manipulation of microalgae regarding the elimination of HMMs, which could be exploited for the development of a sustainable environment. In addition, large-scale phycoremediation processes must be implemented with genetic engineering, immobilization techniques, chemical pre-treatment, and other physicochemical strategies to enable a rational design for the removal of HMMs through microalgae.

## Data Availability

Not applicable.
